# Delayed initiation of anti-retroviral therapy in TB/HIV co-infected patients, Sanyati District, Zimbabwe, 2011-2012

**DOI:** 10.11604/pamj.2015.21.28.5195

**Published:** 2015-05-13

**Authors:** Brian Abel Maponga, Daniel Chirundu, Notion Tafara Gombe, Mufuta Tshimanga, Donewell Bangure, Lucia Takundwa

**Affiliations:** 1 Department of Community Medicine, University of Zimbabwe, Zimbabwe; 2 Department of Health, Kadoma City Council, Zimbabwe

**Keywords:** Tuberculosis, HIV, delay, initiation, anti-retroviral therapy, Sanyati, Zimbabwe

## Abstract

**Introduction:**

Tuberculosis (TB) remains a public health problem and is driven by HIV. Recent studies indicate that anti-retroviral therapy (ART) initiated during the first two months of anti-TB treatment (ATT) reduces risk of HIV morbidity and mortality. In Sanyati district, 14% of TB/HIV co-infected patients were initiated on ART during TB treatment, in 2010. The study was conducted to determine the magnitude and determinants of delay in ART initiation, in TB/HIV co-infected patients.

**Methods:**

An analytic cross sectional study was conducted at three study sites in Sanyati district. The outcome was delayed ART initiation, being failure to be initiated on ART during the first two months of ATT. Respondents were interviewed using pre-tested questionnaires. Epi-Info™ was used to generate frequencies, means, odds ratios and 95% confidence intervals. Stratified and logistic regression analysis was done.

**Results:**

Of the 186 respondents, 63% had delayed ART initiation. Median delay from initiation of ATT to ART was 48 days (Q_1_=20; Q_3_=82). Risk factors for delayed ART initiation were: being treated for TB first time, AOR=2.23 (p=0.03); initially registered for HIV care outside Sanyati, AOR=3.08 (p<0.01); staying more than 5km from a clinic, AOR=3.29 (p<0.01). Enabling factors for early ART initiation was having a family member on ART, AOR=0.23 (p<0.01).

**Conclusion:**

Significant delay and barriers to ART initiation were identified. Decentralization of ART initiation should be expedited. ART initiation should be expedited in patients with identified risk factors for delaying ART initiation. Peer support should be strengthened in families and community. Periodic evaluation of magnitude of delay and impact of early ART initiation in TB/HIV patients is recommended.

## Introduction

Tuberculosis (TB) remains a challenge to global public health. It has been difficult to control in areas with a high prevalence of human immunodeficiency virus (HIV) infection [1]. Globally, 1.1 million of the 8.8 million new cases of TB reported in 2012 were HIV positive. Of the 1.4 million TB deaths, 350,000 were HIV associated [1, 2]. Worldwide, 11 million people living with HIV (PLHIV) are co-infected with TB. The risk of developing active TB in PLHIV is 21 to 34 times compared to persons without HIV [2]. Approximately 82% of patients with TB/HIV co-infection live in sub-Saharan Africa. The Southern African region, including Zimbabwe, has generalized HIV epidemics. The HIV prevalence exceeds 50% in new TB patients [1, 3, 4]. Since 1993, the directly observed therapy short-course (DOTS) strategy has been the key public health intervention used to control TB. The strategy focused on TB case management of sputum smear-positive cases with use of short-course Rifampicin-containing chemotherapy. The DOTS strategy has been effective in most regions of the world. However, it has been comparatively ineffective in countries with a high prevalence of HIV infection [5]. The WHO and the Stop-TB Partnership has published guidelines, a strategic framework, and an interim policy in response to the TB/HIV challenge [5–7]. Strategies to reduce the burden of TB in HIV-infected persons include Isoniazid preventive therapy (IPT), intensified case finding, and infection control. Strategies to reduce the burden of HIV in patients with TB include provision of: HIV testing and counseling; HIV prevention services; cotrimoxazole prophylaxis (CPT); antiretroviral therapy (ART) and; care and support services [5–7]. Zimbabwe has published guidelines for the management of TB/HIV co-infection, based on WHO guidelines [8].

In 2010, 34% of TB patients (2.1 million) were tested for HIV and accessed HIV prevention, treatment and care services, globally. For TB/HIV co-infected patients, 77% and 46% were enrolled on CPT and ART, respectively [1, 2]. Mathematical modeling suggests that a combination of high levels of ART coverage and early ART initiation at high CD4 cell counts may be required to significantly affect population TB control, especially in settings where TB and HIV infection are hyper endemic [9]. Many studies have shown that delayed initiation of ART in TB/HIV co-infected patients is associated with increased HIV related morbidity and mortality. Zachariah R, *et al*, in Thyolo district, Malawi, in 2007, and Joshua T. Schiffer, *et al*, in the United Kingdom, in 2007 demonstrated that ART initiated during the first two months of TB treatment reduced mortality [10, 11]. Sanyati is a district in Mashonaland West Province, Zimbabwe, with 180,000 inhabitants. Approximately 100,000 people reside in Kadoma City, with the remainder staying in rural, farming and mining settings. There are two public hospitals, Kadoma General Hospital, and Sanyati Mission Hospital, in urban and rural areas, respectively. The hospitals are supported by 13 public clinics. The Zimbabwe National TB Control Program (NTP) targeted to have 100% of TB/HIV co-infected patients on CPT, and 60% on ART during anti-TB treatment (ATT), for the 2010 cohorts [12]. For the first three quarterly TB treatment cohorts of 2010, 39%, 36% and 14% of TB/HIV co-infected patents were commenced on ART during ATT nationally, in Mashonaland West Province, and in Sanyati district, respectively. This is despite efforts to improve access to ART through mobile clinics ART clinics, training of health workers in TB/HIV care, availing treatment guidelines, consistent supply of Efavirenz based ART, and strengthened supportive supervision. The objective of this study was to assess the magnitude and determinants of delayed ART initiation in TB/HIV co-infected patients.

## Methods

An analytic cross sectional study was conducted in Sanyati district, Mashonaland West Province, Zimbabwe. The study sites were TB/HIV clinics at Kadoma General Hospital, Sanyati Mission Hospital, and Rimuka Clinic. The study was conducted among TB patients with a confirmed diagnosis of HIV. Patients co-infected with TB and HIV: who had completed the first two months of ATT; who were initiated on ATT in Sanyati district and; willing to participate, were recruited into the study. Patients co-infected with TB/HIV: who had not yet completed the first two months of ATT; who developed TB whilst on ART; who were initiated ART and ATT outside Sanyati district and; not willing to participate in the study, were excluded from the study. Epi Info^™^7 was used to calculate the sample size. Assuming that 14% of TB/HIV co-infected patients had been commenced on ART during TB treatment in Sanyati district, a minimum sample size of 185 was calculated. The three study sites were purposively selected from the 15 health facilities in the district, as they had TB/HIV clinics. Based on district notification information, a minimum of 109(59%), 52 (28%), and 24(13%) were to be recruited from Rimuka clinic, Kadoma General Hospital, and Sanyati Mission Hospital, respectively. Convenient sampling was used to recruit patients as they came for reviews. Data were collected using a pre-tested, interviewer administered questionnaire. Patient treatment records were reviewed. Delay was calculated in days and was defined as failure to be commenced on ART during the first 2 months of TB treatment, in a TB/HIV co-infected patient. Distance to health facility was measured by estimating the distance of the shortest route from the respondents’ home, to the health facility. The cost of transport was obtained by asking the most frequently paid amount, in American dollars, from the respondents’ home, to ART initiation site, two ways. Key informant interviews were conducted on district TB/HIV program managers, using a discussion guide. The data collected were entered into Epi Info^™^7 (CDC 2012). The same package was used to generate frequencies, means, proportions, prevalence odds ratios (POR). Tests for statistical significance were done at 5% level. Stratified analysis and stepwise forward logistic regression were done to check for effect modification and control for confounding. Permission to conduct the study was obtained from the local health authorities. Ethical approval was obtained from the Medical Research Council of Zimbabwe (MRCZ/B/347). Respondents found not on ART were referred for treatment.

## Results

**Figure 1:**
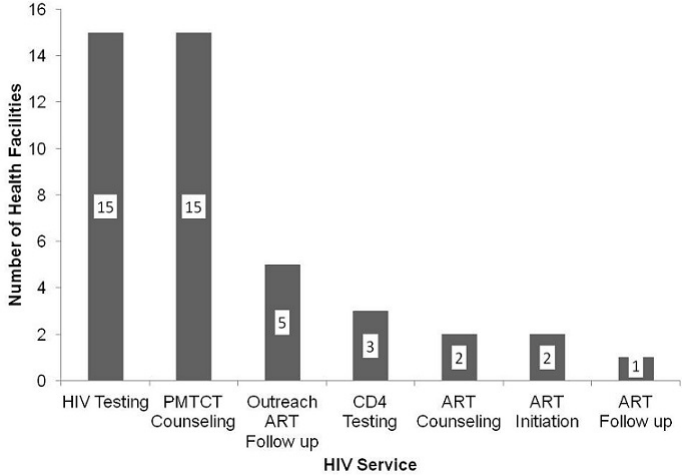
Availability of HIV Care Services in Health Facilities, Sanyati, Zimbabwe, 2012

Key informant interviews indicated that all the 15 health facilities in Sanyati district were providing HIV testing, and counseling for prevention of maternal to child transmission of HIV (PMTCT) prophylaxis. ART counseling and initiation was being provided at the two hospitals. CD4 testing for TB/HIV patients was being done at the 2 hospitals, and clients were charged US$4.00 per test. Figure 1 shows the services that are available for TB/HIV patients, in Sanyati district. One hundred and eighty six respondents were recruited into the study. The respondents were recruited from the study sites as follows: Kadoma Hospital (65%); Rimuka Clinic (20%) and; Sanyati Hospital (15%). The respondents resided in urban (55%), rural (30%), farm (8%) and, mine (7%) settings. Fifty percent of the respondents had initially sought TB/HIV care in public hospitals or clinics. Faith healers (18%), private clinician (17%), traditional healer (6%), herbalist (8%), and pharmacist (1%), were the non public service providers initially visited for TB/HIV care. In terms of TB treatment supervisors, 66% mentioned family member and 31% did not have. The remaining 3% were supervised by community and health centre health workers. Sixty-three percent of the respondents had delayed ART initiation. Table 1 summarizes the delays in processes leading to ART initiation. The overall median delay between ATT initiation and ART initiation was 48 days (Q_1_==20; Q_3_==82). The most delays occurred between HIV testing and first ART counseling (median=19 days, Q_1_=7; Q_3_=56), and between completing ART counseling and ART initiation (median=13 days, Q_1_==0; Q_3_==28). Table 2 summarizes the factors associated with delayed ART initiation in Sanyati district. Patient related factors significantly associated with delayed ART initiation were: staying alone at TB diagnosis (POR=2.84, p=0.01); being a support group member (POR=0.35, p=0.02) and; having a family member on ART (POR=0.24, p=0.00). Service related determinants of delayed ART initiation were: fears of ART drug toxicity (POR=2.19, p=0.03); being treated for TB first time (POR=2.23, p=0.05); having had a CD4 test done (POR=0.24, p=0.00); initially registered for HIV care outside Sanyati (POR=2.95, p=0.01); initially sought TB/HIV care outside public health sector (POR=2.88, p=0.001); staying more than 5 kilometers from health facility (POR=3.51, p=0.002); cost to reach ART initiating site more than US$1 (POR=1.91, p=0.05). On stratified analysis, the type of TB modified the effect of having a CD4 test on delayed ART initiation. EPTB patients who had a CD4 test delayed ART initiation (OR= 2.8, p=0.84). PTB patients who had a CD4 test initiated ART early (OR=0.18, p=0.001), (Chi square for differing OR's =3.98, p=0.046). Independent determinants of delayed ART initiation were: being treated for TB first time (AOR=2.23, p=0.03); initially registered for HIV care outside Sanyati district (AOR=3.08, p=0.01); staying more than 5 kilometers from a health facility (AOR=3.29, p=0.01) and: having a family member on ART (AOR=0.23, p=0.00).

**Table 1:** Delays in Processes Leading to Initiation of ART, Sanyati, 2011-12

Process (number completed process)	Median Delay (Days)	Q_1_; Q_3_ (Days)
Start TB treatment to HIV Testing (186)	-1	-19; 3
HIV Testing to Initiation of ART Counseling (169)	19	7; 56
Initiation to completion of ART Counseling (166)	6	3; 15
Completing ART Counseling to Initiation of ART (n=118)	13	0; 28
Start TB Treatment to ART Initiation (n=118)	48	20; 82

**Table 2:** Determinants of Delayed ART Initiation, Sanyati, 2011-12

Variable	Delayed ART Initiation		POR	p value
	Yes, n=117 (%)	No, n=69 (%)		
Stayed alone at TB diagnosis	38(33)	10(15)	2.84	0.01
Support group member	12(10)	17(25)	0.35	0.02
Family member on ART	40(34)	47(68)	0.24	0.00
Female sex	59(50)	37(54)	0.88	0.79
Urban Residence	60(51)	43(62)	0.64	0.19
Disclosed TB/HIV disease	108(92)	67(97)	0.34	0.31
Perceived stigma community	48(41)	23(33)	1.39	0.38
Perceived Health System Stigma	19(16)	11(16)	1.02	0.88
Feared ART drug toxicity	51(44)	18(26)	2.19	0.03
Treated TB 1^st^ time	16(14)	18(26)	2.23	0.05
Extra Pulmonary TB	15(13)	12(17)	0.70	0.52
Has TB treatment Supporter	75(64)	50(73)	0.68	0.31
Suffered serious illness	39(33)	31(45)	0.61	0.16
On other chronic treatment	17(15)	16(23)	0.57	0.20
Had CD4 test done	75(65)	61(88)	0.24	0.00
Initially registered outside district	39(34)	10(14)	2.95	0.01
Initially sought TB/HIV care outside public health sector	69(59)	23(33)	2.88	0.001
Distance to nearest clinic > 5km	40(35)	9(13)	3.51	0.002
Distance to ART site .10km	52(44)	23(33)	1.60	0.18
Cost to ART initiation site > $1	59(50)	24(35)	1.91	0.005
Was asked for bribe at ART site	4(3)	1(1)	2.41	0.74

## Discussion

In this study the overall delay from initiation of TB treatment to initiation of ART was 48 days. This is shorter than the 67 days delay reported by Gandhi N R, et al, in Sizonq′oba, KwaZulu-Natal, and the 81 days reported in Harare by Dimairo M, *et al*, in 2007 [13, 14]. In Sanyati district, the investments in TB/HIV care might be bearing fruit. However, patient and health system factors may differ between settings. The median delay of 48 days in this study may be an under estimation, as this had been calculated only for 63% of the respondents who had been initiated on ART. The delay in ART initiation is attributed mainly to the delay from HIV testing to initiation of ART counseling (median=19 days) and the delay from completion of ART to initiation of ART by the doctors (median delay=13days). Delay maybe attributed to high ART preparation workload at the two hospitals, as peripheral clinics do not provide ART counseling. Further, only the doctors, in short supply, initiate ART. This is consistent with findings in South Africa by Loveday M, *et al*, in 2011, who reported that shortage of skilled health professionals affects success of TB/HIV care [15]. The main challenge identified in the same study was the policy in use, where only doctors, sometimes not available, initiated ART. In Sanyati district, nurses at peripheral clinics provided PMTCT counseling and ART prophylaxis. This is an opportunity to empower clinic nurses with skills that facilitate decentralization of ART initiation. The availability of psychosocial support and personal experience in TB/HIV care was protective against delaying ART initiation. Having a family member on ART or being a support group member allows sharing of beneficial experience from ART. This may encourage patients to seek HIV care early. In this study, having a treatment supporter was protective. However, the benefit was not statistically significant. This maybe so as the majority (66%) of the respondents had untrained family members as treatment supporters. These untrained family members may not be able to pass the message of the benefit of ART to the TB/HIV clients. Patients who had a CD4 test done, and CD4 count being less than 350 were found to be initiated on ART earlier. This is consistent with the WHO TB/HIV guidelines, where clients with a CD4 count less than 350 should be commenced on ART between two weeks and two months of TB treatment [6, 8]. On stratified analysis, the protective effect of having a CD4 test was beneficial to PTB patients, HIV stage 3 diseases. For respondents being treated for EPTB, stage 4 disease, having a CD4 count delayed ART initiation. This contrasts with the WHO recommendations, where clients suffering from EPTB should be immediately commenced on ART regardless of having a CD4 result [6, 8]. Further, clients without a CD4 test result are likely to delay, as they have to pay US$4 for the test. Some patients may not afford, considering other non medical costs, such as transport.

Respondents who feared ART drug toxicity were more likely to delay ART initiation. Similar results were reported by Kumwenda M, *et al*, in Malawi, 2011, who reported fear of ART drug toxicities and interactions as barriers to ART uptake in TB/HIV co-infected patients [16]. Drug toxicities overlap with those due to TB drugs, and cotrimoxazole, thus the recommended systematic introduction of medications [6, 10]. Potential drug toxicities and the benefits of ART that outweigh potential toxicities should be emphasized during ART preparation sessions. Further, having a relative on ART, and being members of support groups, may help alleviate the fears of ART drug toxicity. Respondents who suffered a serious illness were likely to initiate ART earlier, even though the association was not statistically significant. This is plausible, as the patients are likely to have more contact with the ART initiation hospitals. Further, this could be an indicator of health workers being more compliant in early initiation of ART, to prevent further serious illness. However, the association was not statistically significant, and may have been underestimated due to differences in perception of severity of illness by respondents. Even though most TB/HIV services are for free, efforts to access the centralized ART services, such as cost and distance to reach ART site contributed to delayed ART initiation. However, the distance to reach the ART initiation site was not statistically significant risk factor. This is consistent with findings by Zachariah R, et al, in Thyolo district, Malawi, in 2004, who reported that individuals who had to pay over US$1.00 were not likely to start ART [17]. The trend could be explained by some patients putting up at relatives’ and friends’ places staying near the ART initiation site for preparatory sessions. The association between time and distance with ART delay may also be distorted by variations in estimations, whereas the transport cost is usually reported as a definite value. Thus, efforts to decentralize ART initiation to the peripheral clinics, closer to the patients, may reduce delay. Respondents who stay more than 5 kilometers from a clinic were likely to delay ART initiation. Even though the clinics are not ART initiation sites, however, they provide information on available services, and HIV testing. Hence, those staying further from clinics may need to be reached through innovative measures. This demonstrates a potential gap in information dissemination on TB/HIV care. Thus, innovations, such as use of peer education by support group members, and family members already benefiting from ART may need to be put in place. Clients who initially sought TB/HIV care from outside the public health sector were more likely to delay ART. Similarly, in Zambia, Needham D M, *et al*, 2001, in a study to determine factors associated with delay in TB diagnosis identified having visited private doctor or traditional healer as being a risk factor for TB diagnostic delay [18]. Further, respondents who had initially sought care outside the district delayed ART initiation. Both scenarios demonstrate potential gaps in linkages between service providers, as clients lose time whilst moving places. The study findings may not be generalized to other settings outside Sanyati district. The study design may not have addressed temporality, as exposure and outcome were measured at the same time.

## Conclusion

In this study, delay in ART initiation was significant. Patient and service related barriers to ART initiation were identified. The benefit of peer support amongst patients is demonstrated. Health workers should expedite ART preparation for patients with the identified risks of delay. This can be achieved by reducing the number of ART preparatory visits, and then strengthen counseling during subsequent visits. Emphasis on the benefits of ART that outweigh drug toxicities should be made. TB/HIV patients should be referred to existing support groups for PLHIV. TB/HIV patients who benefited on ART should be used in the families and community to increase awareness on processes in ART and benefits. The health managers should consider reducing the cost of CD4 test, whilst exempting those who cannot afford.
